# Recurrent pulseless electrical activity in a patient with coronary vasospasm and supravalvular aortic stenosis: a case report

**DOI:** 10.1186/s12872-016-0284-5

**Published:** 2016-05-23

**Authors:** Achim Lother, Friedhelm Beyersdorf, Hans H. Osterhues, Christoph Bode, Tobias Wengenmayer

**Affiliations:** Heart Center, Department of Cardiology and Angiology I, University of Freiburg, Hugstetter Str. 55, 79106 Freiburg, Germany; Heart Center, Department of Cardiovascular Surgery, University of Freiburg, Freiburg, Germany; District Hospital Loerrach, Medical Clinic, Loerrach, Germany

**Keywords:** Pulseless electrical activity, Cardiac arrest, Coronary vasospasm, Supravalvular aortic stenosis, ECLS

## Abstract

**Background:**

Pulseless electrical activity cardiac arrest is associated with poor outcomes and the identification of potentially reversible reasons for cardiac arrest is fundamental.

**Case presentation:**

We describe the case of a 46-year-old male with the rare coincidental finding of supravalvular aortic stenosis and coronary vasospasm leading to recurrent pulseless electrical activity cardiac arrest. Extracorporeal life support was successfully applied for hemodynamic stabilization. Supravalvular aorticstenosis underwent surgical repair. The patient survived five time resuscitation and was discharged after full neurological recovery.

**Conclusions:**

Coronary vasospasm and supravalvular aortic stenosis are rare but potentially reversible causes of pulseless electrical activity cardiac arrest. Extracorporeal life support allows accurate diagnostic and possibly therapy even of uncommon reasons for cardiac arrest.

**Electronic supplementary material:**

The online version of this article (doi:10.1186/s12872-016-0284-5) contains supplementary material, which is available to authorized users.

## Background

Cardiac arrest due to pulseless electrical activity (PEA) is associated with poor survival and neurological outcome when compared to a shockable rhythm [1, 2]. To provide successful resuscitation it is fundamental to identify potentially reversible causes for cardiac arrest. The reasons for PEA are considered to be more often non-cardiac including hyperkalemia, hypoglycemia, bleeding or hypovolemia, pulmonary embolism, pneumothorax and cardiac tamponade [2–4]. This is reflected by current treatment guidelines that recommend the use of laboratory testing or ultrasound during resuscitation [3]. However, cardiac disease needs to be considered as an alternative cause of refractory PEA. Extracorporeal cardiac life support is more and more commonly available and can be applied to allow diagnostic and treatment of potentially reversible causes [3]. These include also uncommon findings that might be missed by routine measures during resuscitation.

We describe here for the first time the case of a patient with the rare coincident finding of coronary vasospasm and supravalvular aortic stenosis leading to recurrent pulseless electrical activity cardiac arrests.

## Case presentation

**Fig. 1:**
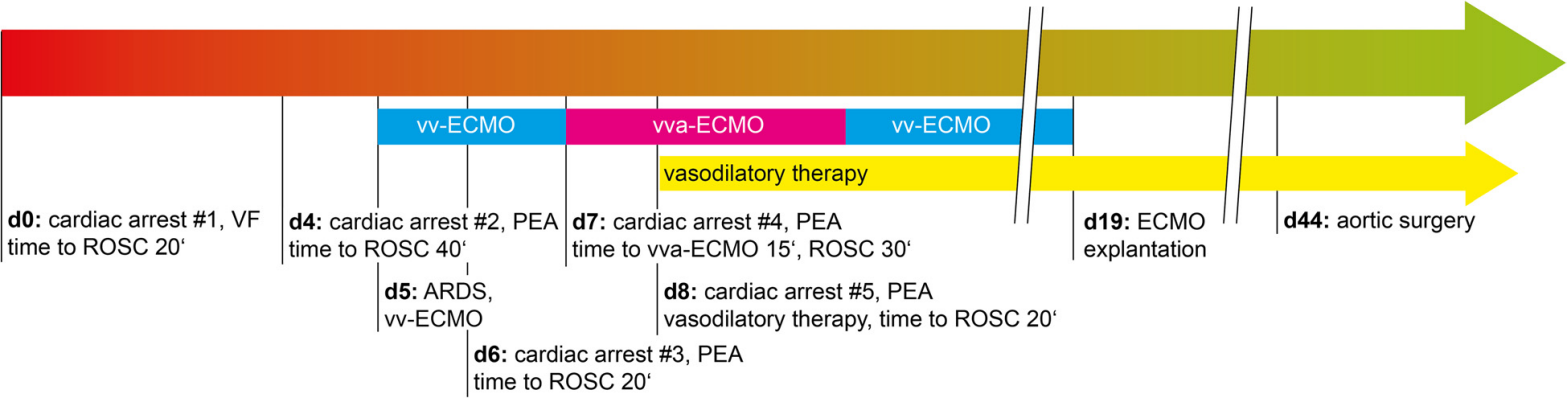
Time course. Schematic representation of relevant events. For cardiac arrests initial heart rhythm and time to return of spontaneous circulation are given. vv-ECMO, veno-venous extracorporeal membrane oxygenation; vva-ECMO, veno-veno-arterial extracorporeal membrane oxygenation; PEA, pulseless electrical activity; VF, ventricular fibrillation; ROSC, return of spontaneous circulation

**Fig. 2:**
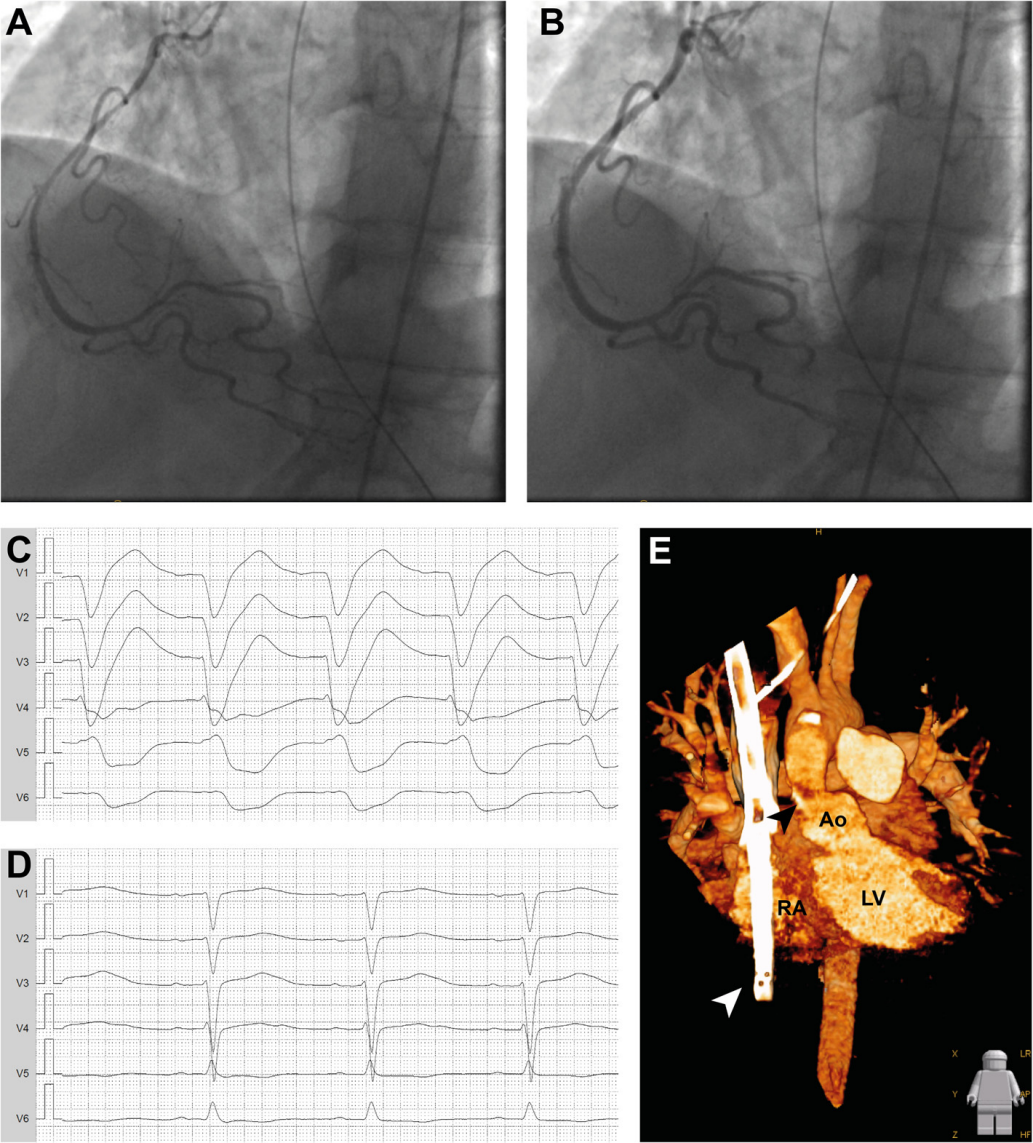
Coronary vasospasm and supravalvular aortic stenosis leading to pulseless electrical activity cardiac arrest. Coronary angiography of the right coronary artery revealing coronary vasospasm (**a**) that was relieved by application of glycerol trinitrate (**b**). ECG recording showing marked intraventricular block during pulseless electrical activity cardiac arrest (**c**) and a restored QRS (**d**) after vasodilatory therapy. 3D reconstruction of computer-assisted tomography (**e**) displaying the supravalvular aortic stenosis (*black arrow*) and double lumen ECMO cannula (*white arrow*). Ao, Aorta; LV, left ventricle; RA, right atrium

A 46-year-old male was successfully resuscitated for out-of-hospital cardiac arrest due to ventricular fibrillation (Fig. 1). Coronary angiography immediately after admission to the county hospital revealed coronary vasospasm but no coronary artery disease (Fig. 2a). After intracoronary administration of glycerol trinitrate the coronary system exhibited regular flow without any relevant stenosis (Fig. 2b). Pulmonary embolism, aortic dissection and other potentially reversible causes were ruled out by computer-assisted tomography (CT) of the chest and lab-testing. Mild therapeutic hypothermia was established for 24 h. For aspiration pneumonia he was treated with ampicillin and sulbactam.

Because of the development of a severe acute respiratory distress syndrome five days after admission our ECMO team established a veno-venous extra-corporeal membrane oxygenation (vvECMO) system using a 31 Fr bi-caval cannula and transferred the patient to our center (Fig. 1). Another CT of the chest showed progressive bi-pulmonary infiltration but no pulmonary embolism. Subsequently sufficient oxygenation was delivered by combined ECMO treatment and lung protective ventilation.

During his stay in the intensive care unit the patient underwent recurrent episodes of cardiac arrest with pulseless electrical activity (PEA, Fig. 1, Fig. 2c) that were not associated with hypoxemia as he was on full ECMO support. There was no evidence for other causes for PEA like tension pneumothorax or embolism in the bedside ultrasonography and echocardiography. Disturbances in electrolytes could be ruled out be point-of-care testing. As we could not reach a return of spontaneous circulation after 15 min of resuscitation we successfully modified the established vvECMO to a veno-veno-arterial ECMO system by introduction of a 17 Fr delivering cannula into the right femoral artery in order to provide extracorporeal life support (ECLS). This procedure could be performed within 15 min without complications under ongoing CPR. During the following episode PEA was successfully terminated when coronary vasospasm was relieved by i.v. administration of glycerol trinitrate and verapamil while circulation was maintained by ECLS (Fig. 1, Fig. 2d). In total, the patient had experienced five episodes of resuscitation till then. A combined long-acting nitrate and calcium channel blocker vasodilatory therapy was established and successfully prevented further events. ECMO therapy could be stopped 14 days after implantation, weaning from mechanical ventilation was successful after 35 days.

The patient has no family history of cardiac death. He underwent patch-repair for supravalvular aortic stenosis during childhood. On day 6 transthoracic and transesophageal echocardiography revealed a remaining supravalvular aortic stenosis (V_max_ 4 m/s, mean pressure gradient 37 mmHg) that was confirmed by CT (Fig.1e, Additional file 1: Video S1). Older medical records showed, that the re-stenosis had been already detected during a rehabilitation exam 20 years ago but did not cause any symptoms. Since hemodynamics remained stable after initiation of vasodilatory therapy surgical repair was postponed until respiratory recovery. The stenosis was successfully relieved by supracoronary ascending aortic replacement on day 44 (Fig. 1).

The patient had full neurological recovery and was discharged to follow-up treatment. A cardiac defibrillator was implanted for secondary prevention of the initial VF.

## Discussion

We report a case of recurrent cardiac arrests due to coronary vasospasm and supravalvular aortic stenosis.

The patient survived five episodes of cardiac arrest, four of them with PEA which is an uncommon complication of vasospastic angina. Patients with vasospastic angina pectoris are at higher risk of sudden cardiac death and most often cardiac arrest occurs due to tachycardic ventricular arrhythmia [5]. Nevertheless, as reported in this case, presentation might be different. There are only few previous reports of PEA associated with coronary vasospasm [5–8], one of them describing recurrent cardiac arrests [6]. The patient described in this case had a supravalvular aortic stenosis which might have a) aggravated low cardiac output during coronary vasospasm and b) triggered coronary vasospasm due to elevated pressures in the aortic bulbus. Supravalvular aortic stenosis is rare in general population but frequent in Williams-Beuren syndrome, a genetic disorder that is associated with an increased rate of cardiovascular events and death [9]. We did not find one of the known Williams-Beuren mutations or other syndromic presentation in this patient.

Veno-arterial ECMO is considered as the preferred method for extracorporeal life support to allow further diagnosis and therapeutic measures when standard life support fails to achieve ROSC [3]. Percutaneous cannulation can be performed fast and with a low rate of complications in the intensive care unit or the cath lab by experienced teams [10]. In this case veno-veno-arterial ECMO was applied to treat concurrent ARDS and cardiac arrests.

## Conclusion

In conclusion, the coincidence of coronary vasospasm and supravalvular aortic stenosis lead to recurrent pulseless electrical activity cardiac arrest in this case. Unusual causes for refractory cardiac arrest should be considered during and after resuscitation. Extracorporeal life support provides the opportunity for accurate diagnostic and therapy when routine diagnostic measures are not sufficient.

## Abbreviations

CT, computer-assisted tomography; ECLS, extracorporeal life support; ECMO, extracorporeal membrane oxygenation; PEA, pulseless electrical activity; ROSC, return of spontaneous circulation.

## Additional file

Additional file 1: Animated 3D reconstruction of computer-assisted tomography displaying the supravalvular aortic stenosis and double lumen ECMO cannula. (MP4 10783 kb)
